# Antibody-Mediated Fcγ Receptor-Based Mechanisms of HIV Inhibition: Recent Findings and New Vaccination Strategies

**DOI:** 10.3390/v1031265

**Published:** 2009-12-15

**Authors:** Vincent Holl, Maryse Peressin, Christiane Moog

**Affiliations:** UMR_S U748 INSERM/UdS, Institute of Virology, Faculty of Medicine, University of Strasbourg, 3, rue Koeberle, F-67000 Strasbourg, France; E-Mails: maryse.peressin@unistra.fr (M.P.); c.moog@unistra.fr (C.M.)

**Keywords:** neutralizing antibodies, FcγR-bearing immune cells, non-neutralizing inhibitory antibodies, mucosal HIV vaccine

## Abstract

The HIV/AIDS pandemic is one of the most devastating pandemics worldwide. Today, the major route of infection by HIV is sexual transmission. One of the most promising strategies for vaccination against HIV sexual infection is the development of a mucosal vaccine, which should be able to induce strong local and systemic protective immunity. It is believed that both humoral and cellular immune responses are needed for inducing a sterilizing protection against HIV. Recently, passive administration of monoclonal neutralizing antibodies in macaques infected by vaginal challenge demonstrated a crucial role of FcγRs in the protection afforded by these antibodies. This questioned about the role of innate and adaptive immune functions, including ADCC, ADCVI, phagocytosis of opsonized HIV particles and the production of inflammatory cytokines and chemokines, in the mechanism of HIV inhibition *in vivo*. Other monoclonal antibodies - non-neutralizing inhibitory antibodies - which recognize immunogenic epitopes, have been shown to display potent FcγRs-dependent inhibition of HIV replication *in vitro*. The potential role of these antibodies in protection against sexual transmission of HIV and their biological relevance for the development of an HIV vaccine therefore need to be determined. This review highlights the potential role of FcγRs-mediated innate and adaptive immune functions in the mechanism of HIV protection.

## 1. Introduction

Currently, one estimates at more than 40 million the number of people living with HIV/AIDS in the world. AIDS is and remains one of the principal causes of death worldwide (more than 33 million people have died from the infection since the discovery of HIV). Each year, two to three million individuals become infected worldwide, corresponding to about 7,500 new cases of infection per day (*Report on the Global AIDS Epidemic* Geneva; UNAIDS; 2008.). The number of people infected has stabilized since 2008, but the HIV/AIDS epidemic cannot yet be considered to be under control. Despite efforts to develop effective anti-retroviral drugs, many infected individuals have no access to treatment with multi-drug regimens. The development of a prophylactic vaccine is, thus, still a matter of priority, if we are to limit the pandemic. Today, the majority of the people infected by HIV are women (in India, Latin America, North America, Thailand, China and East Europe) and the heterosexual transmission of HIV *via* the genital mucosae has become the major mode of infection. In more than 80% of newly diagnosed cases of HIV-1 infection, the patients were infected during sexual intercourse. Currently, one of the innovating vaccination strategies would consist in developing a mucosal vaccine as an effective means of prevention against HIV sexual transmission. Such a vaccine should stimulate the production of particular antibodies, mucosal HIV-specific antibodies (mainly, IgG and secretory IgA) that are able to neutralize free viral particles and to inhibit infection of mucosal HIV target cells before the establishment of systemic infection, in addition to a strong induction of cellular immunity. Such antibodies, by preventing the infection of the first target cells of the virus such as immature dendritic cells and resident macrophages, localized in the vaginal mucosa (epithelium and submucosal sites), constitute a first line of defense against the virus at this portal of entry. These key cells of the anti-infectious immunity are described to be permissive to HIV *in vitro*, being infected *in vivo* and producing *de novo* viral particles [1–4]. Many recent works have highlighted the central role of these antigen-presenting cells (APCs) in HIV pathogenesis. *In vitro*, these dendritic cells have been shown to transfer infectious viral particles to nearby CD4 T lymphocytes in *trans* [3–7]. Cell-to-cell transmission of HIV has been proposed to be a very efficient mode of infection and to participate to the dissemination of the virus throughout the body. It is believed that antibodies, which neutralize HIV infection of these primary target cells, constitute one of the components of the immune response to induce by vaccination. However, only 10 to 20% of the patients develop antibodies able to neutralize a broad spectrum of primary isolates of HIV [8]. These types of antibody are only seldom detected after vaccination in the conventional neutralization assay. After several years of intensive research, only a small number of neutralizing monoclonal antibodies that inhibit a broad spectrum of HIV primary isolates were described to date. The neutralizing activity of these antibodies has been evaluated *in vitro* during the infection of primary blood CD4 T lymphocytes (the principal target cells of HIV) [9] and, more recently, with human cell lines expressing receptor and co-receptor of HIV [10]. Several studies largely showed that the passive transfer of neutralizing antibodies, *i.e*., antibodies able to inhibit the viral replication *in vitro*, make it possible to protect the macaques from an experimental infection with chimerical viruses (SHIV) [11–15]. Recently, it has been demonstrated that Fcγ portion of neutralizing monoclonal IgG1 b12 is crucially involved in the mechanism of HIV protection mediated by passive administration of this neutralizing IgG after vaginal SHIV challenge in non-human primate model: indicating that FcγRs are important in HIV protection by neutralizing antibodies [13]. However, the Fcγ-mediated inhibitory activity of HIV-specific antibodies was not well evaluated *in vitro*; for instance, the activity of HIV-specific antibodies (neutralizing *versus* non-neutralizing IgG) on HIV replication in other human primary target cells such as macrophages [16,17] and dendritic cells [18,19] was been little studied and are poorly understood. Recently, antibodies that differ from neutralizing antibodies, referred to as unconventional antiviral or non-neutralizing inhibitory antibodies (reviewed in [20]) have been described to play a potent role in the inhibition of HIV replication in these APCs [21–23]. These antibodies could represent new additional antibodies to induce by vaccinal immunization. In the present review, certain aspects concerning HIV inhibition by antibodies such as neutralization and Fcγ-mediated inhibitory activity will be discuss, and consequences for the development of new vaccination strategies will be highlighted.

## 2. IgG structure and functions

Antibodies, particularly those of the IgG type, are key mediators of the protective humoral immunity. IgG and other Ig are composed of constant and variable regions: the antigen binding site (Fab) is constituted of the association of variable and constant regions, whereas the so-called Fc domain is formed by two constant regions. Through their Fab parts, antibodies recognized specific epitopes at the membrane surface of pathogen and through their Fcγ domain; they act as immune response modulators, notably by interacting with Fcγ receptors (FcγRs).

### 2.1. Role of Fcγ glycosylation

IgG glycosylation has been shown to play a key role in modulating antibody binding to FcγRs [24,25]. The Fc domain of IgG harbors a sugar moiety, consisting of a conserved biantennary core structure with additional fucose and sialic acid residues [26]. Glycosylation of IgG has been shown to be essential for binding to FcγRs (whether activating or inhibitory) (Table 1). While removal of the whole sugar moiety from the Fc part will change its structural integrity [27], resulting in impaired binding IgG to FcγRs [28], but variations in Fc glycosylation may affect FcγR binding in different ways [29]. For example, low levels of fucose specifically increase the affinity of IgG for activating FcγRIIIa [30], a receptor involved in triggering antibody-dependent cell cytotoxicity (ADCC). Galactose, another residue in the sugar moiety, has also been implicated in IgG activity. Indeed, some autoimmune disorders are associated with increased level of IgG without terminal galactose and sialic acid [28]. Conversely, highly sialylated IgG glycoforms display low FcγRs binding capacity. Thus, IgG enriched with sialic acid had a diminished cytotoxic activity *in vivo*, consistent with their reduced affinity for FcγRs. It was demonstrated that sialic acid confers enhanced anti-inflammatory activity to IgG *via* upregulation of inhibitory FcγRIIb expression [31]. This receptor has been described to be mostly implicated in regulatory function of innate and adaptive immunity [25]. An indirect mechanism has been proposed, suggesting that other unknown receptors exist and are able to sense sialic-acid-rich IgG [24]. Variations in IgG glycoforms *in vivo* have been reported upon immunization [32]. IgG fucosylation increases in response to repeated immunization with ovalbumin, suggesting that changes in IgG fucosylation may be of biological significances. These findings are consistent with the notion that antigen-specific antibodies generated in response to immunization acquire the ability to mediate a protective inflammatory response through switching to an IgG population with lower levels of sialylation. These data open up new possibilities for research aiming to induce the production of specific IgG subsets by immunization against HIV. For example, it would be interesting to analyze the association of IgG subclasses and Fc domain glycosylation with the HIV inhibitory activities of IgG in HIV controllers. In vaccinated HIV individuals, such associations need also to be determined. The role of the Fcγ portion of such antibodies in the immune function need to be investigate and clarify in order to design further HIV vaccine candidates that are able to elicit antibodies with optimal FcγR engagements.

### 2.2. Antigen-specific IgG functions

The antigen-specific IgG specialize in the recognition of pathogenic agents, including HIV-1, and trigger various immune functions, such as neutralization, aggregation, phagocytosis, degranulation, cellular cytotoxicity (ADCC), virus inhibition (ADCVI or antibody-dependent cell-mediated virus inhibition) and the release of pro-inflammatory cytokines or chemokines. These antibodies may mediate either pro- or anti- inflammatory effects, by binding to activating or inhibitory FcγRs. *In vivo*, immune cell activations or functions are triggered by the engagement of specific FcγRs (balanced between activating or inhibitory FcγRs). These activating and inhibitory FcγRs are differentially expressed on a wide range of innate immune effector cells, including basophils, mast cells, neutrophils, monocytes, macrophages, dendritic cells, eosinophils, neutrophils, NK cells, γδ T and B lymphocytes (reviewed in [33]). The binding of immune complexes to FcγRs on dendritic cells or macrophages, for example, has been shown to result in the phagocytosis, degradation and presentation of antigenic peptide on MHC class I and II molecules (leading to the activation of the other antigen-specific immune cells such as CTLs, CD4 T-helper cells, Treg cells, *etc.*) [34]. Other FcγR effector functions of have been also described, such as ADCC and ADCVI for instance. As part of humoral immune responses, ADCC is a mechanism whereby a FcγR-bearing effector cell lysed a target cell that expressed foreign antigens, which have been recognized and bound by specific antibodies. Similar to ADCC, ADCVI will measure both cytolytic and noncytolytic killing of infected cells by FcγR-bearing effector cells. NK cells are widely believed to be the major mediators of ADCC *in vivo* [35,36], but others cells of the immunity, in particular monocytes, macrophages [37], neutrophils, eosinophils and γδ T cells have also been described to mediate ADCC [38]. As the large majority of these cells are present in different sites, more often in peripheral tissues, they can act early during the initial events of the infection. ADCC activity has mainly been attributed to FcγRIIIa and IgG1 and IgG3 isotypes. FcγRIIIa presents at the cell membrane of a subset of NK cells is able to recognize Fc part of IgG which its Fab portion will bound to HIV antigen present at the cell membrane of infected target cells. Once bound to FcγRIII, NK cells released cytokines such as IFN-γ, and cytotoxic granules containing perforin and granzymes that promote cell death by triggering apoptosis of the targeted cells. FcɛRI of eosinophils and monocytes can also induce cell death by recognizing IgE-bound cancer cells [39,40].

In the case of HIV vaccine development, optimizing HIV-specific antibody activities by enhancing their interaction with FcγRs would likely to improve the viral fate and disease outcome. It might be useful for example to enhance HIV-specific IgG reactions such as antibody-mediated destruction of virus-infected cells at mucosal site of viral entry. Moreover, improving HIV antigen presentation by using antibody as targeting molecule for the delivery of antigen to specialized APCs *via* FcγRs would not only enhance viral degradation but also boost specific antiviral immunity. All these points will need to be addressed during the development of an effective HIV vaccine.

## 3. Broadly neutralizing antibodies against HIV

Despite intensive research in the field of HIV vaccine development over the last 20 years, only a small number of monoclonal antibodies that display highly active broadly neutralizing activity have been discovered. These antibodies include five well known neutralizing monoclonal antibodies capable of strongly inhibiting the replication of a large spectrum of primary HIV isolates and protecting macaques against SHIV infection. Three of these monoclonal antibodies (IgG1 b12, 2G12 and 447-52D) are directed against specific epitopes of gp120 and the other two (2F5 and 4E10) are directed against the membrane-proximal external region (MPER) of gp41 [41–45]. More recent studies, based on the use of high-throughput strategies for screening and identifying the neutralizing activities of antibodies from HIV patients, have led to the discovery of new additional broadly neutralizing monoclonal antibodies [46–48].

### 3.1. Monoclonal neutralizing IgG : IgG1 b12, 2G12, 447-52D, 2F5 and 4E10

IgG1 b12 was the first neutralizing antibody to be identified. This antibody recognizes the CD4 receptor-binding site on gp120, which is conserved between various HIV subtypes [39]. IgG1 b12 showed an unusual protuberance of his paratope that confers such specificity, in particular, to plunge in the cavity of the gp120 responsible for the attachment to CD4, inhibiting by this fact the binding of HIV to its host receptor. Many other antibodies recognizing epitopes in the vicinity of the CD4 binding site have been found in HIV-infected individuals, but none of them exhibited potent neutralizing activity [49]. 2G12 recognizes an epitope based on the C4/V4 region of gp120 [50–53]. Conserved mannose residues on the distal end of the oligomannose glycan located around the base of the V3 and V4 loops in the outer and “silent” face of gp120 are required for 2G12 binding. This epitope is located on carbohydrate residues, which are generally poorly immunogenic or even tolerogenic, as HIV used host glycosylation machinery. 2G12 is a very interesting and unique antibody, as its structure is unconventional and differs from the classical “Y”-shaped form of IgG antibodies in that the two heavy structures of the arms are vertically adjacent. This specific configuration enables this antibody to display neutralizing activity against many HIV clades [54]. Thus, consistent with the apparently unique nature of 2G12, such neutralizing antibodies are difficult to elicit. It has been proposed that this antibody may prevent HIV attachment to the CD4 receptor and to alternative receptors, such as DCSIGN (by competing with 2G12 for binding to gp120) [50,55], thereby inhibiting non-specific HIV fusion.

447-52D recognizes 14 amino acids, including the conserved stretch of four amino acids - GPGR - core sequence at the tip of the hypervariable V3 loop [50]. The conservation of this sequence at the tip of the V3 loop is directly related to the function of this loop in binding to the HIV coreceptor. 447-52D neutralizes primary isolates bearing the GPGR V3 motif, regardless of whether these viruses belong to clades A, B, F, or H. However, this sequence is more highly conserved among subtype B viruses. Independently of the CXCR4 and CCR5 coreceptor usage, this neutralizing antibody is able to neutralize both R5- and X4-HIV strains. The interaction of 447-52D with the V3 loop seems to be unique, as it is not observed with other anti-V3 antibodies. The V3 region of some HIV primary isolates is somewhat inaccessible to 447-52D, due to the presence of glycan moieties or V1/V2 regions, often rendering these viruses resistant to neutralization. Some resistant viruses become sensitive to 447-52D neutralization after binding to their receptors. Thus, conformational changes may expose critical epitopes, making them accessible for antibody recognition. Such observation has also been made for neutralizing CD4 induced antibodies that need HIV binding to its receptor for recognizing other neutralizing masked epitopes.

Monoclonal neutralizing 2F5 and 4E10 recognize a conserved epitope of the HIV gp41 that has been directly implicated in viral fusion. 2F5 binds to an epitope involving a linear amino acid motif ELDKWA on the gp41. These antibodies cannot prevent HIV attachment to the cells, but a later phenomenon of virus-host cell membrane fusion [41]. They were obtained from EBV-immortalized B-cell clones generated from PBMCs from various HIV-infected subjects [42]. These antibodies have been described to have lipid polyreactivity and CDR3 motifs suggestive of autoimmune antibodies [56,57]. Their neutralizing activity is mediated by the long CDR3 H3 loop that penetrates deeply into the antigen cleft [49]. This has led to the suggestion that immune dysregulation may contribute to the development of these MPER antibodies [56,57], accounting for the rarity of 2F5- and 4E10-like antibodies in HIV patients.

### 3.2. New broadly neutralizing antibodies: recent findings

By examining neutralization breadth in sera or plasma samples from large cohorts of infected individuals, new broadly cross-neutralizing antibodies have been recently discovered [46–48]. Using a single round of infection of TZM-bl cells with pseudo-virus particles, as the main neutralization assay for the detection of neutralizing antibodies, the group of Lynn Moris has demonstrated that various subclasses of IgG directed against a novel epitope within the gp41 MPER have potent heterologous neutralizing activity. These antibodies detected in three broadly neutralizing plasma of chronically infected individuals, and for one (BB34) of them, were mainly from the IgG3 subclass. Originally, 4E10, 2F5 and neutralizing fraction of a HIVIG were all of the IgG3 subclass [58,59]. IgG3 antibodies appear early in infection and have a very flexible hinge region that is thought to render the MPER region highly accessible to these antibodies. However, class switching from IgG3 to IgG1 did not affect the neutralizing activity of the well-known neutralizing 2F5 and 4E10 antibodies, indicating a lack of requirement of the IgG subclass for MPER-mediated neutralization. Interestingly, like 2F5 and 4E10, the anti-MPER neutralizing activity detected in BB34 was potentiated by the presence of FcγRI on TZM-bl, indicating that these new recognized epitopes within the MPER region may confirm the relevance of such immunogens for rational vaccine design [60], as FcγRs have been shown to play a crucial role in the mechanism of protection by neutralizing antibodies in macaques [61]. Further evaluations are required to determine whether the subclass (IgG1 or IgG3) of these neutralizing antibodies affects their potency *in vivo*, due to differences in Fcγ affinity for the corresponding FcγRs. Although, it has been proposed that such anti-MPER antibodies are autoreactive and therefore eliminated through B-cell tolerance mechanisms [56], these data showed that neutralizing anti-MPER antibodies are seldom detected in HIV-infected individuals. The presence of anti-MPER antibodies in broadly neutralizing blood samples has also been reported [62,63]. These neutralizing antibodies that target MPER epitopes overlapping the 2F5 epitope were not produced during the acute phase of infection but rather 12 to 27 months following HIV transmission and coincident with the development of auto-antibodies. Indeed, others have concluded that the time period following HIV exposure is an important parameter that could influence neutralization breadth and antibody avidity [64]. The antibody activity or potency to neutralize HIV may also depend of the maturation of its Fab domain [65]. Others may assume that post-translational changes in antibody structure would turn a non-neutralizing antibody into a broadly neutralizing one or enhance the cross-neutralizing activity of weak neutralizing antibody. Due to poor immunogenicity of these neutralizing epitopes, we need to identify new cross-neutralizing antibodies that would recognize immunogenic epitopes, which are more accessible on HIV envelope to facilitate vaccine design.

The group of Dennis Burton has recently isolated, from an African donor, two potent monoclonal antibodies (PG9 and PG16). These antibodies were unable to bind monomeric gp120 and gp41 while they neutralize native viral particles. They recognized a new cross-neutralizing epitope expressed on the quaternary envelope structure of the conserved region of V2/V3 loops [48]. Their gene analysis revealed that these broadly neutralizing antibodies had a long CDR3 loop, typical of polyreactive antibody. The reactivity of these antibodies against several antigens was tested and no polyreactivity was found. Interestingly, these neutralizing antibodies display different neutralizing activities against any given virus. These neutralization data undoubtedly reflect the capacity of the immune system to evolve with viral envelope mutations. As these new neutralizing antibodies display broad neutralization activities, particularly, against non-clade B HIV primary isolates, they would provide protection against the most prevalent HIV variants worldwide if they could be induced by vaccination.

### 3.3. *In vivo* production of neutralizing antibodies: proof-of-concept

A recent study has demonstrated that the *in vivo* administration of an adeno-associated virus (AAV) vector carrying the gene encoding an engineered neutralizing antibody (distinct from IgG1 b12 and 2G12) can induce the production of the biologically active neutralizing antibody in the serum of macaques and maintained a long-term memory immunity [66]. In the vaccinated monkeys, neutralizing antibodies produced *in vivo* can protect them from an intravenous SIV challenge, at virus inoculum concentrations known to infect 100% of the macaques [66]. Such systems have been previously been used to generate cross-neutralizing IgG1 b12 production in mouse serum, as a proof-of-concept [67]. In addition, memory antibody responses to HIV have recently been characterized, in particular from infected patients with low viral loads and broadly neutralizing antibodies [68], suggesting that HIV-specific humoral immune responses can achieve such neutralizing antibody production. These results pave the way for innovative new approaches for the cloning of neutralizing antibodies from HIV individuals. Altogether, these data provide new hope for the rational design of new vaccine candidates for eliciting broad-range cross-neutralizing antibodies.

## 4. Neutralizing IgG as a hallmark of protection against HIV

The particular hallmarks of humoral immunity to HIV have raised many questions about the role of neutralizing antibodies during HIV infection *in vivo*. In rhesus monkeys, the reduction in the number of B lymphocytes delays the appearance of neutralizing antibodies after infection with SIVmac251, but has no effect on the initial control of the viral load during early phase of primo-infection [69]. Moreover, in non-progressor HIV-patients a strong humoral response made up of neutralizing antibodies has been detected associated with the control of viremia [70–72].

### 4.1. Neutralizing antibodies as immune correlates of protection

Now, there is widespread acceptance that eliciting neutralizing antibodies is likely to be an important component of an effective HIV-1 vaccine [73]. The neutralizing activity of antibodies is largely considered as the mainstay of antiviral immune protection mediated by Ig. It is well known that *in vitro* HIV-1 neutralization by monoclonal IgG is correlated with *in vivo* protection against HIV-1 in macaque model [12,13,61,74–76]. Several years ago, it was clearly demonstrated that the passive administration of a mixture of neutralizing antibodies prevents the vaginal transmission of pathogenic chimeric virus (SHIV) in rhesus macaques [12]. Mascola *et al.,* have reported that high-doses of broadly neutralizing antibodies provided an effective protection against mucosal HIV transmission [12]. Following the passive transfer of neutralizing antibodies, an analysis of mucosal secretions showed that these antibodies were able to transudate in the mucosal compartments (nasal, oral and vaginal mucosa), suggesting greater protective activity against the infection of macaques *via* the vaginal route [12]. Baba *et al.,* also showed that the passive transfer of neutralizing IgG to new-born primates protects against oral SHIV transmission, suggesting a role for these neutralizing IgG in the inhibition of vertical HIV transmission from mothers to infants [11]. Another report showed that the passive transfer of the monoclonal neutralizing antibodies 2F5 and 2G12, which have broad neutralizing activity against primary HIV isolates [77], together with a purified polyclonal IgG obtained from the plasma of HIV patients, prevented intravenous SHIV infection in macaques [13]. Several other studies have also described that the passive administration of neutralizing monoclonal antibodies to non-human primates before or after viral challenge can protect the animals against infection [78]. Nishimura *et al.,* showed that the passive transfer of neutralizing antibodies one hour following the infection of the primates, rather than 24 hours, conferred an increased effectiveness of protection [15]. The presence of these neutralizing antibodies before the establishment of infection seems to be the key determinant of the efficacy of antibodies. The initial studies in macaques highlighted the role of such neutralizing IgG in mucosal protection, but the use of progesterone to thin the cervico-vaginal surfaces may improve the transudation of these antibodies into mucosal fluid, thereby conferring a higher level of infection. Therefore, it has been speculated that, in the absence of hormone treatment, higher concentrations of systemic neutralizing IgG may be needed for efficient mucosal protection. However, this notion has been recently reconsidered, following the report from Ann Hessell *et al.,* that low doses of neutralizing 2G12 antibodies protected rhesus macaques against experimental mucosal challenge [55]. This protection does not seem to be due to higher levels of 2G12 transudation or transport from blood to the vaginal mucosal surface following intravenous antibody administration in macaques. The authors also determined the capacity of 2G12 to induce ADCVI and showed that 2G12 promoted ADCVI less efficient than IgG1 b12. Nevertheless, Fcγ-mediated inhibitory activities, other than ADCVI or ADCC, including phagocytosis and the clearance of immune complexes, were not evaluated. It therefore remains possible that 2G12 may compensate its neutralizing activity by other “extra-neutralizing activities”. Taken together, these data strongly suggest that higher doses of neutralizing antibodies are not necessary to confer a sterilizing protection in macaques and that other mechanisms of HIV protection, different from the neutralization, are certainly involved [79]. On the other hand, it has been suggested that the vaginal inoculation of macaques with large amounts of virus is not representative of the physiopathological conditions underlying sexually transmitted infection. Mostly, sexual HIV infection in human has occurred after multiple exposures to the virus, suggesting that repeated administration of low doses of virus inoculum in non-human primates may be the most appropriate model for studying the capacity of HIV-specific antibodies to prevent mucosal infection. Another recent report from the group of Dennis Burton suggested that the repeated passive administration of low levels of monoclonal neutralizing IgG1 b12 antibodies was sufficient to protect macaques against the repeated low-dose exposure of SHIV to the mucosal surfaces of the vaginal walls [76].

### 4.2. Mechanism of protection-mediated by neutralizing IgG: role of FcγRs

Dennis Burton and his group have recently demonstrated that neutralizing monoclonal IgG1 b12, in the absence of Fc-FcγR functions, cannot efficiently protect macaques from vaginal infection, indicating that FcγRs are crucially involved in the mechanism of antibody protection against vaginal SHIV challenge [61]. Given to these data, it is the most evidence that FcγRs are important in the mechanism of protection-mediated by neutralizing antibodies. When analyzing HIV neutralization on monocyte-derived macrophages and dendritic cells, an enhancement of the neutralizing activity was also detected for the five well-known monoclonal neutralizing IgG [21], but not for their corresponding neutralizing Fab fragments [21,60], indicating that the Fc portion of the these IgG as well as the FcγRs are strongly implicated in this increased neutralization [21,22]. Of note under these conditions, a higher neutralizing or inhibitory activity of neutralizing 2F5 and 4E10 than of IgG1 b12 was detected, suggesting that the conserved epitopes on gp41 are more relevant than the CD4 binding site epitope for neutralization of macrophages infection. Much like these findings, recently Perez *et al*., also observed that the neutralization activities of both monoclonal neutralizing 2F5 and 4E10, and, other plasma samples from chronically HIV-infected blood donors containing gp41 MPER-specific antibodies were potentiated by FcγRI and, to a lesser extent, by FcγRIIb [60]. *In vitro*, these recent data have also emphasized the role of FcγRs in the mechanism of HIV inhibition by neutralizing IgG. As another example, previous studies have also confirmed that the passive transfer of monoclonal antibodies directed against West Nile virus cell surface-associated proteins triggers Fcγ receptor-mediated phagocytosis and the clearance of infected cells and thus protection [80–82]. Altogether, these previous studies highlight the potent role of the Fc moiety of the IgG in the *in vivo* protection and *in vitro* inhibition afforded by the neutralizing antibody and raise questions about the effector functions induced by their cognate FcγRs present at the cell surface of specific immune cells.

As proof-of-concept, all these numerous passive transfer studies of neutralizing monoclonal antibodies have shown that administration of broadly reactive antibodies in non-human primates can afford sterilizing protection from vaginal or systemic infection [11–13,15,61,74,75,83], demonstrating the potential role of virus-specific humoral immunity [78]. In an important way, the recent studies in non-human primates also emphazised that FcγRs play a crucial role in the mucosal protection against infection by neutralizing antibodies [61].

## 5. Non-neutralizing inhibitory IgG against HIV as antiviral antibodies

### 5.1. Role of non-neutralizing IgG in HIV infection

Unlike neutralizing antibodies, non-neutralizing antibodies, which are found in many HIV patients, are induced during the acute phase of HIV infection and generally before the induction of cross-neutralizing antibodies. They may be generated by immunization or vaccination. Their role in HIV infection *in vitro* and *in vivo* was poorly understood or is somewhat controversial. Conversely, several groups have reported an FcγRs-mediated antibody-dependent enhancement (ADE) of infection *in vitro*, which they attributed to the action of non-neutralizing antibodies [84–89]. Takeda *et al*., showed that subneutralizing concentrations of antibody-positive sera increased HIV replication in the U937 human monocytic cell line [84], whereas optimal antibody concentrations did not. Others have shown that by blocking FcγRIII, but not FcγRI or FcγRII, inhibit the enhancement of HIV infection in peripheral blood macrophages [85,86]. By contrast, FcγR-independent ADE of HIV infection in MT2-CD4^+^ T lymphoblastoid cells has been detected in a small proportion of serum samples from HIV-seropositive individuals [90], suggesting that there may be several modes of ADE. One paper to date has described the ADE of HIV infection in FcαR-expressing cells [91]. Complement-dependent ADE has also been observed in certain types of dendritic cells (reviewed in [92]).

Preliminary studies have reported that the neutralizing activities of polyclonal IgG purified from the plasma or sera of SIV-infected macaques or HIV-infected patients are increased by a factor of 100 to 1,000-fold when monocyte-derived macrophages instead of blood mononuclear cells are used as target cells of virus [16,17]. Interestingly, they have reported that some purified IgG samples from the sera of recently infected individuals (a few months after seroconversion) or from recently infected macaques (six months after SIV inoculation) have potent neutralizing or inhibitory activities on macrophages and not on PBMCs. This suggests that non-neutralizing antibodies, which are produced before neutralizing antibodies, may play a role in the early stages of infection. More recently, it has been reported that non-neutralizing antibody activities were correlated with the reduction of acute viremia in vaccinated macaques [93], demonstrating that non-neutralizing antibodies-elicited by immunization could play an important role in the control of the acute phase of infection mediated, at least in part, by ADCVI activity and transcytosis inhibition.

### 5.2. FcγRs-mediated HIV inhibition by antibodies: role of non-neutralizing inhibitory IgG?

Although a role of the FcγRIII in the mechanism of HIV inhibition by antibodies, independently of the CD4 receptor, has been described in macrophages [86], we [21] and, more recently, the group of David Montefiori [60], have definitively demonstrated that human FcγRs are involved in the inhibition of HIV replication by some HIV-specific IgG in human blood monocyte-derived macrophages and in transformed TZM-bl cells expressing various FcγRs, respectively. Others have shown that the activation of human macrophages, through FcγRs cross-linking, can contribute to the natural protection against HIV infection observed in some HIV-exposed uninfected individuals by inhibiting mucosal virus transmission [94]. Some time ago, the team of Michael Fanger evaluated the role of FcγRs in the infectivity of monocytes and macrophages, using bispecific antibodies to target HIV particles to various FcγR-bearing cells; they found that these FcγRs were not involved in the ADE of HIV infection in these cells [95].

*In vitro* some non-neutralizing IgG (monoclonal or polyclonal antibodies from HIV-1 patients) strongly inhibit HIV replication in macrophages or dendritic cells generated from human blood monocytes [21–23]. In addition, it has also been demonstrated that HIV infection and provirus formation are impaired in immature dendritic cells in the presence of HIV-specific IgG and complement [96]. The long-term transfer of HIV from dendritic cells to CD4 T cells also seems to be impeded by these HIV-specific IgG, whereas the addition of these antibodies and/or complement to activated PBMC did not protect them from HIV infection. It has thus been concluded that unconventional mechanisms of HIV inhibition by such antiviral antibodies may take place in dendritic cells but not in PBMC, leading to the suggestion that the lower levels of infection mediated by HIV-IgG immune complexes may result from interactions of virus-bound IgG with FcγRIIb expressed on DCs [96].

The Fab domain and the Fc constant domain of IgG and FcγRs present at the membrane of these APCs are involved in the efficient inhibition of HIV-1 replication in these immune cells by monoclonal and polyclonal non-neutralizing inhibitory IgG. More interestingly, we have reported that some monoclonal non-neutralizing IgG recognized specific epitopes on the viral gp, particularly in the V3 loop regions of gp120 and certain domains of gp41 (e.g., the immunogenic principal immunodominant domain) [23]. For some of them, their inhibitory concentrations that reduced 90% of infection are of similar magnitude that those of monoclonal neutralizing antibodies when these FcγR-bearing immune cells are used as HIV targets cells. These non-neutralizing inhibitory antibodies possess unconventional antiviral activities on FcγR-bearing immune cells. Such antibodies (some nonneutralizing inhibitory IgG as well as the monoclonal neutralizing IgG1 b12 for example) are not detected in the new neutralization assay performed with FcγR-expressing TZM-bl cells [60], undoubtedly due to differences in viral epitope-antibody recognition or host-cell susceptibility to HIV infection (high levels of expression of CCR5) or that these cell lines express only one of each FcγR subtype and not a combination of multiple FcγRs.

For example, it has been reported that the level of CCR5 expression, but not of CD4, on cell surface of HeLa cells may modify the neutralization efficiency of certain 4E10-like neutralizing antibodies [97]. These unconventional inhibitory IgG, despite being unable to neutralize free viral particles in the conventional neutralization assay, display potent FcγR-mediated HIV inhibition in other unconventional assays [20]. Effectively, the Fcγ portion of these anti-HIV antibodies may bind to the corresponding FcγRI or II on the cell surface of these APCs, thereby mediating HIV inhibition by a mechanism involving the phagocytosis and clearance of HIV-IgG immune complexes. This mechanism of HIV inhibition, distinct from the neutralization of the infectious viral particles by the Fab part of the antibody, exploits FcγR functions induced by IgG.

Other non-neutralizing antibodies recognizing epitopes present on the V2 loop, the C5 constant region or the CD4 binding site of HIV cannot prevent HIV replication in APCs [23]. Recently, using cord blood CD34-derived Langerhans and interstitial dendritic cells as HIV target cells, we confirmed that FcγRs are involved in HIV inhibition by neutralizing as well as non-neutralizing inhibitory antibodies (personal communication, unpublished results). No antibody-dependent enhancement of HIV replication has been detected in this *in vitro* inhibitory assay when macrophages or dendritic cells are used as HIV target cells. These data support the view that other types of HIV inhibition mechanism are implicated, and, point out that new assays need to be set up to understand the underlying mechanism of action.

Moreover, Donald Forthal and teammates have shown that NK cells and the Fc part of polyclonal IgG are required for the enhanced HIV-1 inhibition in PBMCs [98]. They also recently published that recombinant gp120 vaccination can elicit antibodies with antiviral activity against clinical strains of HIV-1, which necessitate the presence of FcγR-bearing effector NK cells [99]. They found that these antibodies inhibited HIV infection in the presence of effector cells, such as NK cells or Fcγ-bearing cells, whereas F(ab’)_2_ fragments of these antibodies did not protect permissive cells against infection more effectively than whole IgG. A correlation has been found between the level of ADCVI and the gene polymorphism of FcγRIIa and FcγRIII.

**Figure 1.**
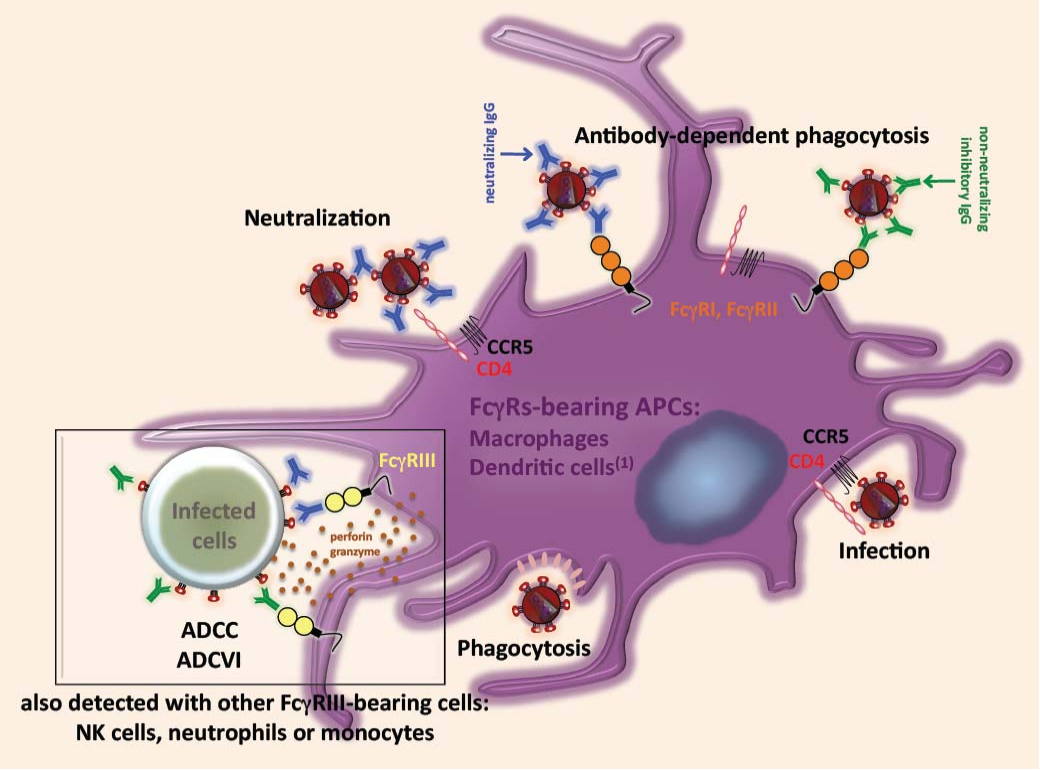
Mechanisms of antibody-dependent FcγRs-mediated HIV inhibition. A schematic overview of the role of FcγR-bearing cells, such as macrophages or immature dendritic cells, in the mechanism of HIV inhibition by neutralizing and non-neutralizing inhibitory IgG. Macrophages and dendritic cells located at the portal of HIV entry, and other immune cells present in the blood, such as NK cells may play an important role in preventing the establishment of HIV infection. Some macrophages and dendritic cells can degrade free-viral particles by phagocytosis for antigen presentation, but these cells are also permissive to the virus and can produced *de novo* viral particles. Due to the expression of FcγRs, including FcγRI and II in particular, at the cell surface of these cells, neutralizing and non-neutralizing inhibitory IgG can inhibit HIV infection by a mechanism of antibody-dependent phagocytosis leading to viral degradation in an endolysosomal compartment within these antigen-presenting cells (APCs). In addition, FcγRIII may play a role in the killing of infected cells by an ADCC-based mechanism. Moreover, FcγRI has been reported to mediate ADCC by neutrophils. ^(1)^ There is no clear evidence that dendritic cells could mediate ADCC, unlike they express low levels of FcγRIII. Besides, this FcγR has also been reported to inhibit viral replication by a mechanism of ADCVI mediated by other cells, such as NK cells, monocytes and neutrophils. This figure illustrates the key role played by FcγRs in the mechanism of HIV inhibition by neutralizing antibodies and by some specific antiviral antibodies.

In addition to the neutralizing activity of antibody, Fc-FcγR interactions are essential for *in vivo* protection against HIV infection. For these reasons, antibody-mediated effector mechanisms other than neutralization are clearly of great importance in protection against HIV [100]. The mechanisms by which FcγRs can enhance the HIV inhibitory activities of such antibodies remain unclear. However, FcγR-bearing immune cells involved in first-line defenses against mucosal HIV transmission are likely to play an important role in the clearance of opsonized virus by specific antibodies (Figure 1). The *in vivo* relevance of such non-neutralizing inhibitory or antiviral antibodies mediating FcγR effector functions remains to be clarified.

### 5.3. HIV inhibition by antibody-dependent cell cytotoxicity (ADCC)

Several studies have shown that ADCC is one mechanism through which HIV-specific antibodies can limit or control viremia during HIV infection; such antiviral antibodies are often detected in the plasma of acutely infected patients [101–105], whereas neutralizing antibodies are generally absent. Unlike neutralizing activity, ADCC activity is detectable in most of HIV-infected individuals within a few days after seroconversion, and is correlated with the decline of the viremia [104]. ADCC activity can also be involved in disease progression, as non progressors, unlike rapid progressors, have high levels of ADCC antibodies [106]. Other HIV-infected individuals with undetectable levels of virus also have higher titers of such antibodies than viremic subjects [107]. Similarly, ADCC activity has recently been detected in HIV controllers at significantly higher levels than in viremic individuals [108]. In macaques, ADCC has also been correlated with delayed progression to AIDS, confirming the previous observations in humans [109,110]. Dysfunction NK cell activity has been reported in HIV-infected individuals [111]. The NK cells of infected patients produce IFN-γ but are unable to mediate ADCC *in vitro* [112]. In infected patients treated by HAART, a subset of blood cells expressing NK markers have been reported to display persistent HIV infection [113], suggesting that NK cells may serve as a reservoir for the virus. Other studies have also reported that HIV may modify the ADCC function of macrophages and neutrophils [114]. To date, various ADCC assays have been developed to analyze more precisely the activity of such antibodies to kill HIV infected cells, as reviewed in [115]. More recently, as HIV-specific ADCC antibodies triggered multiple effector functions of NK cells upon FcγRIII cross-linking (e.g., IFN-γ, TNF-α and β-chemokines production, degranulation of perforin and granzymes, CD107a expression, *etc.*)[116], new assays have been setup to monitor directly the activation status of the NK cells. According to the literature, all these compounds have antiviral activity and can directly interfere with HIV infection or infected cells.

Several ADCC epitopes on HIV have been identified, some of which are present in many HIV-infected individuals [114]. V3 loop ADCC epitopes appear to be the major determinants for ADCC activity, but ADCC epitopes in the V2 domain of gp120 have also been defined. It seems likely that the presence of ADCC epitopes in a variable region may result in viral variants escaping ADCC responses. Thus, analyzing of viral escape from ADCC activities would identify a subset of conserved ADCC epitopes. No clear correlations between vaccine-induced ADCC responses and *in vivo* protection have yet been observed, but most of the available evidence suggests that ADCC responses control HIV infection.

The role of ADCC in vaccination has been evaluated. Mucosal prime systemic boost immunization of macaques can elicit both ADCC and neutralizing antibodies, but protection against mucosal SHIV challenge is correlated with the titers of neutralizing antibodies, rather than with those of ADCC antibodies [114,117]. Others studies have reported that titers of human rgp120 vaccine-induced FcγR-dependent antibodies, which inhibit HIV infection *in vitro*, are inversely correlated with the infection rate [99]. It is thus important to determine which types of HIV-specific ADCC antibodies are required to prevent HIV infection and the likelihood of their induction by vaccination. It is well known that non-neutralizing antibodies, that mediated ADCC responses, can potentially contribute to mucosal defense against HIV, if present in cervico-vaginal secretions [118,119].

### 5.4. Antibody-dependent cell-mediated virus inhibition (ADCVI)

Like ADCC, antibody-dependent cell-mediated virus inhibition or ADCVI results from an interaction between an infected target cell, a HIV-specific antibody and an effector cell expressing one or several FcγRs. Unlike ADCC, ADCVI reflects the impact of antibody and effector cells on viral replication [61,99,104,120]. ADCVI encompasses multiple effector function activities related to lytic (e.g., ADCC) and non-cytolytic (e.g. production of β-chemokines, *etc*.) mechanisms dependent on FcγRs that may interfere with HIV infection and replication. *In vivo*, the ADCVI activity of nonneutralizing antibodies may help to reduce viral load and is strongly correlated with Fc-FcγR-mediated HIV inhibition by neutralizing antibodies *in vitro* [55,61]. ADCVI may be a reliable indicator of the antiviral activity of non-neutralizing antibodies that are not detected in conventional neutralization assays.

## 6. Neutralizing IgA as mucosal immunity against sexual HIV transmission

Twenty years ago, it has been described that HIV-specific immunity could be detected in antibody-positive and antigen-negative sexual partners of known HIV-seropositive individuals [121,122]. Since, intensive investigations of the epidemiologic, genetic and immune characteristics of this minority group of individuals, either women or men, such as sexual partners of many different HIV-infected persons, commercial sex workers, healthy newborns of HIV-infected mothers or some drug users, who remained uninfected despite repeated exposure to HIV, have been carried out by several international HIV research groups. The observation of resistance to HIV infection in these highly HIV-exposed individuals has attracted the curiosity of the scientific community over the years. For many reports, the lack of infection in these individuals may be associated with the exclusive priming of T lymphocytes [122]. Subsequent reports showed that a particular genetic background or the presence of soluble HIV inhibitor factors, or cytotoxic T lymphocytes and a potent NK cells activity can be detected in mucosal fluids, potentially accounting for this resistance to HIV infection [122].

The presence of HIV-specific IgG and IgA has also been examined in the cervico-vaginal samples and plasma of HIV-exposed uninfected subjects (mainly women from Italy, India, Kenya, Thailand, Cambodia, *etc.*) *versus* infected people. Several studies have shown that HIV-specific IgA were present in the genital tract of some HIV-uninfected heterosexual partners of seropositive individuals [123–130]. These subjects have been referred as highly sexually exposed to HIV-1 but persistently IgG seronegative or HEPS. The presence of mucosal IgA has been observed in several but not all groups of HEPS subjects [131,132]. HIV-specific IgA has also been detected in the seminal fluid of male seronegative partners of HIV-positive women [133]. Moreover, in some infants of infected mothers, HIV-specific salivary IgA have been observed [134]. It has been proposed that the CCL28 chemokine may preferentially attract and increase the rate of IgA-secreting plasma cells in the epithelial lamina propria, thereby increasing the amount of specific IgA produced. By contrast, in many HIV-infected persons, HIV-specific IgG and IgA were found in plasma and mucosal samples. None of the HIV-resistant sex workers had HIV-specific systemic and mucosal IgG responses. The absence of detectable HIV-specific IgG in the sera of these HEPS remains puzzling, because of the conventional view of Ig class-switching. This discrepancy may result from technical issues relating to the detection of IgA in mucosal fluids and the methods used for the collection of these fluids. In 10% of the HIV-exposed uninfected female sex workers, HIV-specific mucosal IgG were detected in the absence of mucosal viral antigen-specific IgA were detected [132]. However, these uninfected women had no detectable HIV-specific IgG in their plasma. These findings suggest that some uninfected female sex workers may have developed low-level mucosal IgG responses against HIV. In some circumstances, the frequency of genital tract IgA may be underestimated with the respect to that of IgG due to antibody competition for HIV binding sites [135] and the mucosal environment (local bacterial infection, IgA1 protease activity, genetic differences, *etc.*). In such cases, co-infected individuals may have a transient IgA deficiency at the mucosal surface, without being susceptible to the acquisition of HIV-infection. HIV-specific Th responses have been associated with HIV resistance in some cases, in several cohorts of HEPS patients [124,136,137]. However, these cellular responses are not correlated with HIV-specific mucosal IgA responses, consistent with systemic cellular and mucosal humoral responses being independent mechanisms of protection against HIV infection. The genital mucosae are enriched in lymphoid follicules specializing in the development of IgA-producing plasma cells. Thus, mucosal HIV-specific IgA immunity may rapidly become estalished locally, resulting in the containment of HIV infection (thus preventing IgG seroconversion), independently of host cellular responses. It has been proposed that the signaling pathway required for systemic IgA responses, which seem to be dependent on the germinal center, are strikingly different from those of mucosal IgA responses.

It has recently been shown that the presence of mucosal monoclonal IgA antibodies specific for HIV gp41 is a correlate of protection in some HEPS individuals. These HIV gp41-specific IgA have been characterized: they are able to neutralize HIV and inhibit HIV transcytosis. Their Fab domain has a specificity similar to that of the broadly neutralizing monoclonal IgG antibodies 2F5 and 4E10 [138]. Polyclonal HIV-specific IgA purified from the sera of asymptomatic HIV patients has been shown to display HIV neutralizing activities, as detected in the conventional neutralization assay using peripheral blood leukocyte population as HIV target cells [8]. As for these polyclonal neutralizing IgA, the neutralizing titers of these gp41-specific IgA from HEPS patients are closed to those of neutralizing IgG 2F5, indicating that the presence of low doses of these neutralizing IgA in genital secretions may be active *in vivo* [138]. Relative to the specificity of epitope targeted by these IgA, the recognized region of gp41 seems to be in conformational configuration and conserved between clade B and C HIV strains. As antibody maturation is a process of great importance for the neutralizing activity of IgG, one may expect that HEPS individuals, which are subjected to repeat unprotected intercourse and frequent exposure to HIV over a period of months or even years, might therefore be expected to develop potent affinity-matured IgA with neutralizing activity. The HIV seronegativity of these HEPS women is correlated with the production of large amounts of neutralizing IgA in the genital tract and a systemic proliferative response in circulating blood leukocyte populations [125]. These data highlighted the possible involvement of these both independent types of immunity in the protection against sexual HIV transmission.

## 7. Conclusions and Future Directions

In the world, HIV is transmitted mainly by sexual ways. Like other sexually transmitted diseases, HIV infects individuals through the sexual mucosae, particularly in the female genital tract, which constitutes one of the major portals of viral entry. Thus, the induction of protective mucosal immunity is an important goal of future HIV vaccine design. The production of HIV-specific antibodies that are able to prevent sexual HIV transmission is a major challenge for HIV vaccine development. As neutralizing antibodies have been shown to inhibit HIV replication *in vitro* and have been correlated with protection against sexual HIV infection *in vivo*, the induction of such antibodies by vaccination is of prime importance. The development of an effective vaccine able to induce the production of such antibodies would undoubtedly be the best solution for the control of the worldwide AIDS pandemic. None of the various prototype of HIV-1 vaccine candidates designated to elicit humoral and/or cellular immunity has yet afforded protection against infection or decreased viral load after infection [139]. Excepted for the Thai Phase III HIV vaccine clinical trial, it is the first demonstration that a safe and effective AIDS vaccine candidate provides partial benefit in humans. Today, we need to understand why this vaccine regimen worked. After the failure of the Merck HIV vaccine, these results give new hope for the development of a future protective vaccine against HIV.

Many efforts have been made to understand the mechanism underlying the induction of neutralizing antibodies, but so far, they have been largely unsuccessful. One may assume that resolving the mechanism of development of such antibodies will provide critical guidance for pertinent new vaccine strategies. Eliciting neutralizing antibodies by immunization is already one of the main goals of the vaccine research. Current approaches based on AAV encoding gene transfer of neutralizing antibodies have made it possible to induce, *in vivo*, an efficient humoral immune response specific for HIV. The results obtained have important implications for future research aiming to develop new methods of immunization.

For the design of new vaccination strategies or approaches, including mucosal vaccination in particular, we need to determine the role of the specific immunity (production of neutralizing IgA) of cervico-vaginal tissue and its relation with the systemic immunity needs to be clarified. The precise role of HIV-specific neutralizing IgA in the prevention of mucosal HIV transmission needs further investigation; in particular to evaluate if passive infusion of such antibodies could protect from vaginal challenge in non-human primate model. The recent findings that the repeated passive administration of low doses of cross-neutralizing antibodies can confer protection in macaques repeatedly infected vaginally with low levels of viral inoculum has overturned previous notions that high titers of such antibodies are required to generate sterilizing protection against HIV infection.

The demonstration that FcγRs play a crucial role in the neutralizing antibody-mediated protection in macaques has indicated that the Fc portion of such antibody is essential in this protection and not only the neutralizing Fab part. These data have helped to clarify the role of FcγRs in antibody-mediated HIV protection and to upgrade our overall understanding of how neutralizing antibodies work and how they can be induced by immunization. They have also highlighted the need to develop new types of antibody with a higher Fc-FcγR interaction affinity, to increase the efficacy of protection against infection.

The detection of antiviral or inhibitory activities of some non-neutralizing IgG antibodies has highlighted the need to develop new neutralization assays and raises the question of the relevance of these antiviral antibodies in the protection *in vivo*. Not only neutralizing antibodies are highly important in rational vaccine research and design, non-neutralizing inhibitory antibodies, which mediated FcγRs-dependent mechanism of HIV inhibition, should be one of the important components to take into account for further development of novel immunogens to include in future vaccinal preparations.

## Figures and Tables

**Table 1.** IgG glycosylation and effector functions. Human IgG are glycoproteins with a sugar moiety attached to the constant Fc region. The IgG-Fc region is a homodimer consisting of an inter-chain disulfide-bonded hinge region and glycosylated C_H_2 domains bearing an N-linked oligosaccharide at Asn_297_ (as shown in the figure). The sugar residues are shown in color and form the core structure oligosaccharide. Arrows indicate enzymatic cleavage sites used to generate various truncated glycoforms. This glycan core is essential for maintaining a stable and functional Fc structure, a prerequisite for IgG-mediated FcγR effector functions. However, variations in the composition of the sugar moiety may influence Fc-FcγR interaction and activity. Thus, the nature of antibody glycosylation may alter IgG binding to activating or inhibitory FcγRs. Not all Fc-FcγR interactions are equally affected by changes in IgG-Fc glycosylation. IgG-Fc glycosylation may regulate activating and inhibitory Fcγ effector functions, thereby modulating immunity. This table summarizes the influence of the IgG glycosylation on the FcγR effector functions [26–29].

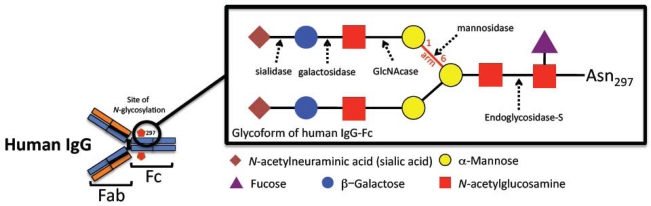	
**Variations in IgG-Fc glycoform**	**Fcγ effector binding and functions**
Complete deglycosylation of IgG1:	- Loss of FcγR binding capacity - Failure to initiate effector function
Removal of fucose residues:	- Increase in IgG affinity (all subclasses) for FcγRIIIa - Enhancement of Fc-FcγR interaction
Removal of galactose residues:	- Lower levels of C1q binding to IgG-Fc - Either increase or decrease in IgG affinity for FcγRs
Exposure of N-acetylglucosamine residues after galactose removal:	- Increase in MBL binding - Observed in some autoimmune diseases
Fc glycan hydrolysis between two N-acetylglucosamine residues:	- Inhibition of the binding of IgG and C1q to FcγRs - Abolition of the activation of effector and complement functions - Impairment of structural stability and functional activity
Truncated glycoforms:	- low IgG binding affinity to FcγRIIb
Increase of the levels of sialic acid:	- Decrease in FcγRIII/IV affinity - No change in IgG binding to FcγRIIb
Replacement of amino acid residues interacting with galactose or N-acetylglucosamine in the 1–6 arm position:	- No influence in IgG binding to FcγRI
Replacement of Asn_297_:	- Loss of N-linked glycosylation - FcγRI affinity halved
Removal of mannose residues in the 1–6 arm position:	- Impairment of FcγRs recognition by IgG
Different glycosylation pattern induced by mammalian cell culture systems:	- Effects on the biological activity of humanized monoclonal antibody - Optimization of effector function activity
Sialylated IgG:	- Anti-inflammatory properties
Aberrant IgG-Fc glycoforms:	- Congenital disorders
